# A Review of Published Analyses of Case-Cohort Studies and Recommendations for Future Reporting

**DOI:** 10.1371/journal.pone.0101176

**Published:** 2014-06-27

**Authors:** Stephen J. Sharp, Manon Poulaliou, Simon G. Thompson, Ian R. White, Angela M. Wood

**Affiliations:** 1 Medical Research Council Epidemiology Unit, University of Cambridge School of Clinical Medicine, Cambridge, United Kingdom; 2 École Nationale de la Statistique et de l’Administration Économique Paris Tech, Paris, France; 3 Department of Public Health and Primary Care, University of Cambridge School of Clinical Medicine, Cambridge, United Kingdom; 4 Medical Research Council Biostatistics Unit, Cambridge Institute of Public Health, Cambridge, United Kingdom; University of Michigan, United States of America

## Abstract

The case-cohort study design combines the advantages of a cohort study with the efficiency of a nested case-control study. However, unlike more standard observational study designs, there are currently no guidelines for reporting results from case-cohort studies. Our aim was to review recent practice in reporting these studies, and develop recommendations for the future. By searching papers published in 24 major medical and epidemiological journals between January 2010 and March 2013 using PubMed, Scopus and Web of Knowledge, we identified 32 papers reporting case-cohort studies. The median subcohort sampling fraction was 4.1% (interquartile range 3.7% to 9.1%). The papers varied in their approaches to describing the numbers of individuals in the original cohort and the subcohort, presenting descriptive data, and in the level of detail provided about the statistical methods used, so it was not always possible to be sure that appropriate analyses had been conducted. Based on the findings of our review, we make recommendations about reporting of the study design, subcohort definition, numbers of participants, descriptive information and statistical methods, which could be used alongside existing STROBE guidelines for reporting observational studies.

## Introduction

**Figure 1:**
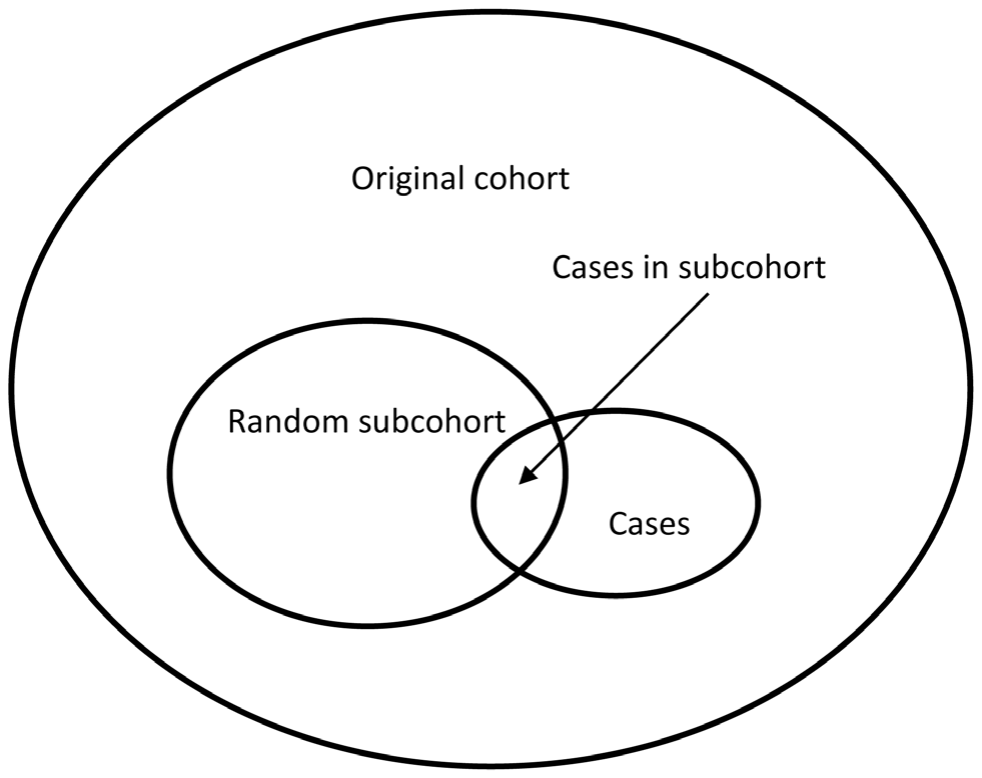
Pictorial representation of an unstratified case-cohort study design. Included in the study are a subcohort of individuals randomly sampled from the original cohort, together with all incident cases of the disease of interest. Because the subcohort is a random sample from the whole original cohort, it includes some incident cases. The subcohort sampling fraction is the proportion of individuals in the original cohort who are included in the random subcohort, and is defined at the start of the study.

The case-cohort study design was originally proposed by Prentice [1]. Nested within a larger cohort, the study comprises a random “subcohort” of individuals from the original cohort (sampled irrespective of disease status), together with all cases [Figure 1]. The main advantage of the case-cohort study design over a cohort study is that full covariate data are only needed on the cases and subcohort individuals, not all the original cohort, potentially saving time and money if measures such as biomarkers or genotypes are required. An advantage of a case-cohort study over a nested case-control study is that the same random subcohort can be used as the comparison group for studying different diseases, rather than identifying a new set of controls for each disease. Also, the process of obtaining measurements on baseline samples from individuals in the random subcohort can be initiated at any time after the original cohort has been set up, whereas in a nested case-control study the cases need to be identified before the controls can be defined and the measurement process begin.

To make inferences from a case-cohort study, it is necessary to account for the over-representation of cases in the sample. Cox proportional hazards (PH) regression models need to be weighted, with cases outside the subcohort only included in the risk set at the time of their event [1]. Different weighting methods have been described in detail [2] and compared by simulation [3]. The usual standard error estimates from the Cox PH model are not valid in the weighted versions, and should be replaced by alternatives such as a robust jack-knife estimator [4]. Weighted Cox regression models can be fit using standard statistical software packages, including Stata [5] and R [6] **.**

The STROBE (Strengthening the Reporting of Observational Studies in Epidemiology) Statement is a checklist of 22 items [7] which provides guidance to authors on the reporting of three types of observational study design: cohort, case-control and cross-sectional studies. However, there is currently no published guidance for case-cohort studies. The aim of this work is to review recent practice in reporting of case-cohort studies, and make recommendations to improve the consistency and quality of reporting these studies in the future.

## Materials and Methods

### Search Strategy

We used the electronic search engines PubMed, Scopus and Web of Knowledge to identify papers reporting analyses of case-cohort studies published between January 2010 and March 2013. We restricted the search to 24 major general medical and epidemiological journals/databases [Appendix S1]. We searched paper titles and abstracts for the keywords “case-cohort” and “case cohort”.

For each paper, we identified the original cohort from which the case-cohort study was constructed, and recorded the number of individuals in the following groups: original cohort, subcohort, total cases, subcohort cases and subcohort non-cases. Where the information was available, we recorded these numbers both before and after exclusion of individuals due to application of specific eligibility criteria for the analysis (e.g. exclusion of individuals with missing values of particular covariates). We recorded whether or not the subcohort was selected by stratified sampling and the stratification factors if it was. For papers where the information was available, we calculated the subcohort sampling fraction as the ratio of the reported size of the subcohort to that of the original cohort, using values before any exclusion criteria were applied. We noted which of the groups of individuals (as defined above) were described using summarized baseline characteristics. We recorded the statistical methods used (and choice of weights, if applicable), whether statistical modelling assumptions were tested, and how missing data were handled in the analysis.

Initial assessment of all papers was carried out by one assessor (MP) and a random selection of 20% of the papers was appraised independently by a second assessor (AW). All discrepancies were resolved by discussion between the two assessors.

## Results

### Papers included in review

We identified 47 published papers using our search strategy. Fifteen papers were excluded from the review for the following reasons: used the term “case-cohort” incorrectly to describe the study they were reporting (N = 9 papers), reported a meta-analysis of published case-cohort and case-control studies (N = 1), used case-cohort analysis methods even though the data included were not from a case-cohort study (N = 1), discussed specific methods for the design and analysis of case-cohort studies (N = 3), described the protocol for a planned case-cohort study (N = 1). The remaining 32 papers (list of references in Appendix S2) were published in eight of the 24 journals/databases considered, with 15 papers published in PLOS ONE. Within the journals covered by this review, the number of published papers reporting case-cohort studies increased between 2010 and 2012 (2010∶5, 2011∶9, 2012∶13), with five papers already published in the first three months of 2013.

### Initial cohorts on which case-cohort studies were based

**Figure 2:**
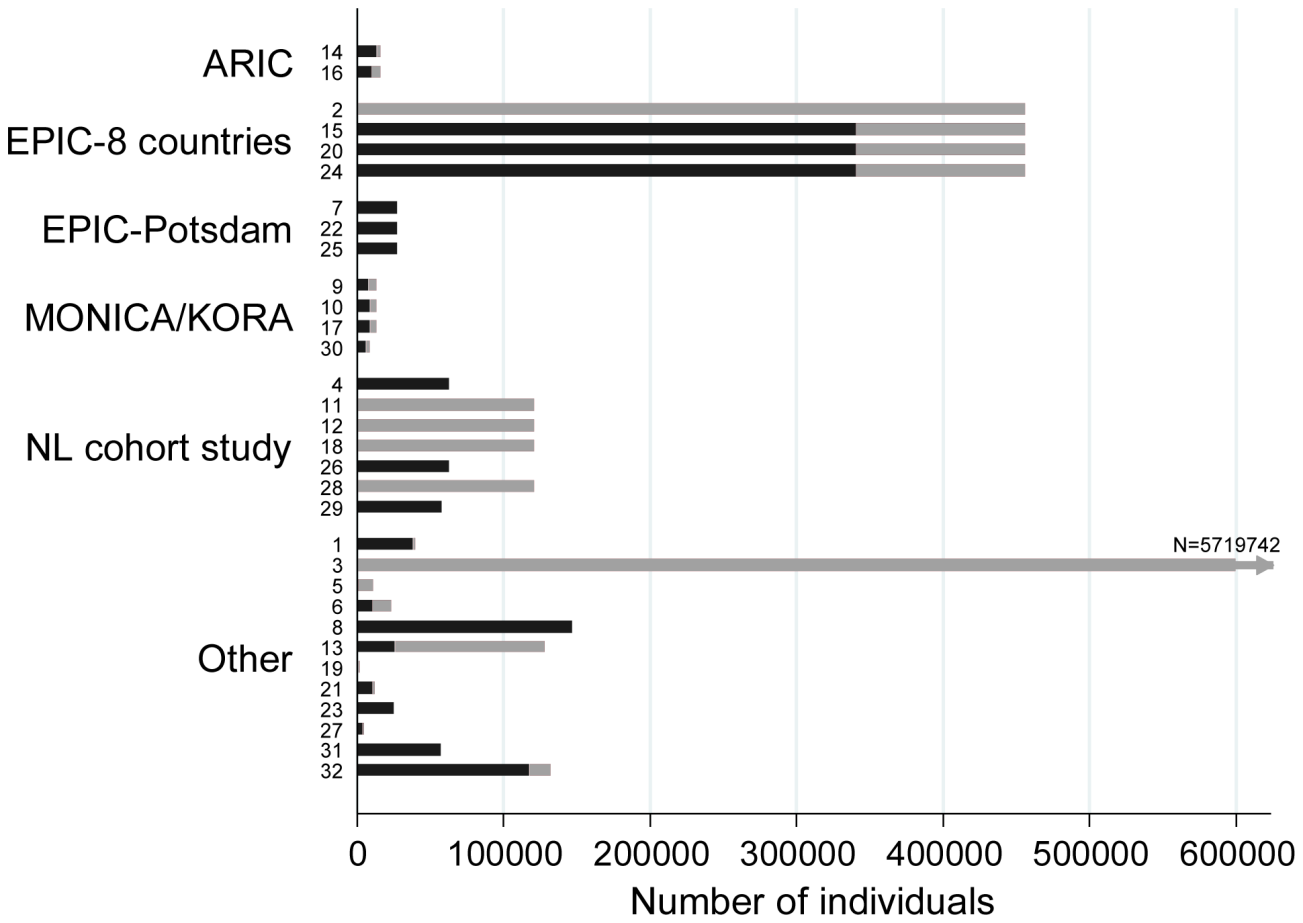
Number of individuals in original cohort on which case-cohort study is based, before and after exclusions. Total length of bar represents number before exclusions, length of black bar represent number after exclusions. Where the bar is all black, the size of the original cohort after exclusions was not reported. Where the bar is all grey, there were no exclusions from the original cohort. Bars are labelled according to the number of the paper in the reference list in Appendix S2.

ARIC, EPIC (8 countries), EPIC-Potsdam (one of the centres within EPIC), MONICA/KORA and the Netherlands cohort study were each the original cohort for more than one paper [Figure 2]. Three individual EPIC-Europe centres and two other groupings of EPIC centres were each the original cohort for exactly one paper. Treating each EPIC centre or grouping of centres as a separate cohort, the 32 papers were based on 17 original cohorts. The sizes of each original cohort before and after exclusions (where reported) are shown in Figure 2. The median size of original cohort before exclusions was 48532 (interquartile range 14610 to 124426). In six papers the size of the original cohort after exclusions was not reported.

### Subcohort sampling

**Figure 3:**
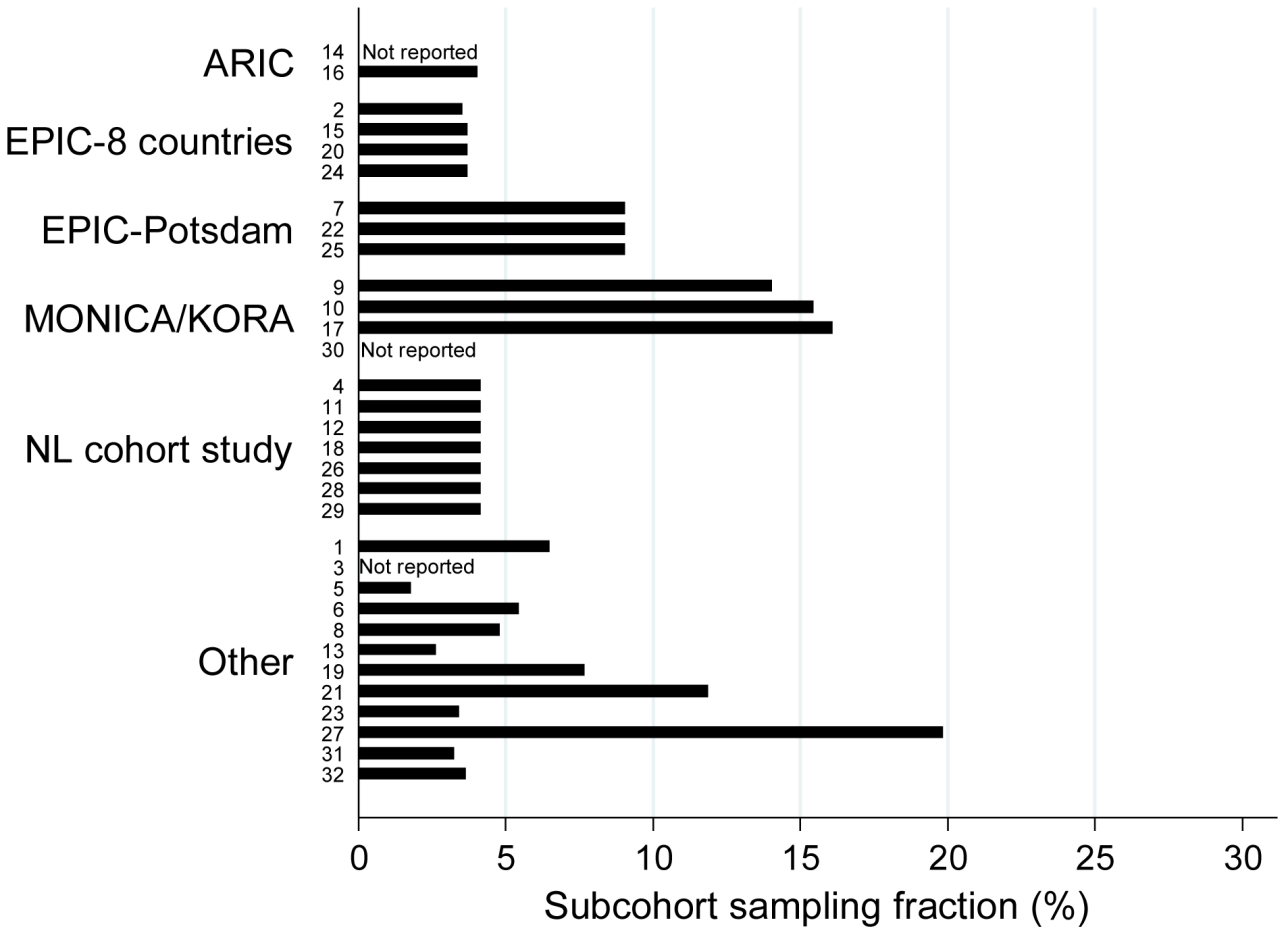
Subcohort sampling fractions reported in each of 32 papers included in the review. Bars are labelled according to the number of the paper in the reference list in Appendix S2.

The median subcohort sampling fraction was 4.1% (interquartile range 3.7% to 9.1%) [Figure 3]. The subcohort sampling fraction was similar, but not always identical for case-cohort studies based on the same original cohort [Figure 3], which suggests that in some of the papers, exclusion criteria had already been applied to either the original cohort or the subcohort, without these being mentioned in the paper.

All papers provided a reference to another publication describing the original cohort, and stated that the subcohort was a random sample from the cohort. Nine of the 17 original cohorts used stratified sampling to select the subcohort. The stratifying variables were age, gender, race, centre or a combination of these.

### Summarizing baseline characteristics

The papers varied in the groups within which baseline characteristics were summarized, and also whether results of statistical comparisons of characteristics between groups were presented [Table 1]. There was a similar number of examples of summaries within cases/subcohort (11) and cases/non-cases (9); none of 11 papers presented any statistical comparisons between cases and subcohort, while seven of nine papers did present statistical comparisons between cases and non-cases. Where characteristics were summarized within exposure groups, this was most commonly done within the subcohort (nine papers); there were four examples of statistical comparisons being performed between exposure groups. Five of 32 papers provided some quantitative summaries of the characteristics of the original cohort from which the subcohort had been sampled.

**Table 1:** Approaches to summarizing baseline characteristics in 32 papers identified in the review.

Groups of individuals in which baseline characteristics were summarized	Number of papers	Statistical comparisons?	
		Yes	No
Cases/subcohort	11	0	11
Cases/non-cases	9	7	2
Subcohort	3	N/A	N/A
Original cohort	5	N/A	N/A
By exposure group within subcohort	9	2	7
By exposure group within cases	2	1	1
By exposure group within original cohort	1	1	0
Baseline characteristics not presented	1	N/A	N/A

Some papers used more than one approach and therefore contribute to more than one row of the table.

### Estimating association between exposure and outcome

All except one paper used some form of Cox regression model to estimate the association between the exposure and disease [Table 2]; the other paper used logistic regression. Of the 31 papers using Cox regression, nine used age as the underlying timescale rather than time in study. Twenty papers specified that a weighted Cox model was used, with 10 using Prentice weights [1] and seven using Barlow weights [2]; in the other three papers it was unclear which weights had been used. One paper applied an extrapolation approach to recreate the original cohort from the case-cohort sample. Seventeen papers specified that robust standard errors were calculated and 12 reported that the PH assumption was tested. Kaplan-Meier plots of cumulative survival or cumulative incidence functions were presented in five papers, although in two of these papers it was unclear whether estimation of these functions had taken the case-cohort design into account.

**Table 2:** Approaches to analysis reported in 31 papers identified in the review that used Cox regression.

Description of method		Number of papers
Choice of timescale	*time in study*	22
	*age*	9
Analysis model	*Cox weighted*	20
	*Cox unweighted*	11
Weights used in Cox model	*Prentice*	10
	*Barlow*	7
	*not stated*	3
Robust standard errors used		17
Proportional hazards assumption		12

### Further aspects of analyses

The four papers based on the EPIC (8 countries) cohort and two papers based on other groupings of EPIC centres, where the subcohort sampling was stratified by centre, described a two-stage approach to reflect the stratified design: first centre-specific models were fit to the data, and second meta-analysis methods were applied to combine the estimates of association across centres. For this second step, five papers used random effects and one used fixed effects meta-analysis.

All the case-cohort studies had individuals with missing values of baseline covariates which were relevant to the analysis being performed; in 27 papers these individuals were excluded from the analysis, while in five papers there was an attempt to include them. In one paper, individuals with missing baseline covariates had their baseline redefined as the first visit with complete data. Four papers described imputation approaches either for the primary or sensitivity analysis.

## Discussion

In this paper we have identified important variability and areas for improvement in the reporting of case-cohort studies in major general medical and epidemiological journals, all of which would be expected to have rigorous statistical review policies. It seems likely that there could be a greater degree of variability and lower quality of reporting in journals with less intensive statistical scrutiny. As with all reviews of this type, deficiencies in reporting do not necessarily imply that the analysis approaches used were inappropriate; however, the findings suggest that some guidance on minimum requirements for reporting these studies could be helpful to authors, journal reviewers and editors. Below, we highlight key aspects of the design and analysis of these studies which should be reported to enable readers to assess the appropriateness of the analyses; these recommendations could be used alongside existing STROBE guidance for reporting observational studies [7].

### Recommendations

#### Study design

Having indicated that a paper is reporting results from a case-cohort study, the original cohort study on which the case-cohort study is based should be described and/or referenced. The case definition, methods of case ascertainment, and dates of start and end of follow-up should be provided. The method for selecting the random subcohort and any exclusion criteria that were applied to the analysis, should be stated. If the sampling was stratified, the stratification factor(s) and rationale for using a stratified design should be provided.

#### Participants

The numbers of ascertained cases and individuals in the random subcohort should be stated both before and after any exclusion criteria have been applied. The number of individuals in the original cohort should also be provided, ideally both before and after application of the same exclusion criteria. If the size of the original cohort after applying exclusion criteria is unknown because the criteria include, for example, excluding individuals with missing data on a variable that is only measured in the case-cohort sample, then this should be stated explicitly. The subcohort sampling fraction should be presented. If the design was stratified, all the above information should be provided within each stratum. The rationale for choosing a particular sampling fraction should be explained, and reasons given if it differs between strata.

#### Descriptive information

Characteristics of study participants, including information on exposures and potential confounders, should be summarized in the usual way using either means and standard deviations, medians and interquartile ranges, or numbers and proportions depending on the type and distribution of the variable. There are various possible groupings of individuals in a case-cohort study for which characteristics could be summarized. If the purpose is to identify variables that are associated with the outcome (i.e. being a case), then characteristics should be summarized in (1) all cases and (2) either all subcohort individuals or all non-cases (i.e. the subcohort excluding cases). If the purpose is to identify variables that are associated with the exposure of interest, then characteristics should be summarized in the subcohort within groups defined by the exposure (groups based on either standard pre-defined cut-offs or quantiles of the exposure distribution in the subcohort). Descriptive information should be presented for the sample included in the analysis after application of exclusion criteria. Consistent with existing STROBE guidance for observational studies [7], significance tests should be avoided in descriptive tables.

It can be helpful to present some descriptive information (where available) for participants in the original cohort from which the random subcohort was sampled, to enable readers to judge the generalizability of the findings and also to assess the extent to which the subcohort used in the analysis is truly representative of the original cohort.

#### Statistical methods

The statistical methods used to estimate the association between exposure and outcome should be stated; for a case-cohort study, methods should appropriately account for the oversampling of cases in the study design. If weights have been used (e.g. for weighted Cox regression), then the weighting method and rationale for its choice should be given. In particular, if Barlow weights have been used, the subcohort sampling fraction should be stated explicitly, since the inverse of the sampling fraction is used to weight subcohort non-cases and cases in the subcohort before they become a case [2]. If the sampling fraction has been calculated as the subcohort size after exclusions as a proportion of the original cohort before exclusions, then the potential impact of using this value in the Barlow-weighted analysis should be explored in sensitivity analyses, which should be described and discussed.

If some form of Cox regression model has been used, the proportional hazards assumption should be assessed for each covariate in the analysis. Appropriate methods for assessing this assumption include fitting and testing interactions between covariates and the underlying analysis timescale, or using a correlation test based on Schoenfeld residuals [8]; an extended version of the Schoenfeld residuals test has been proposed for weighted Cox models [9].

If a stratified sampling design has been used, then a description of how the stratifying factor(s) was accounted for in the analysis should be given. Potential approaches include stratifying the baseline hazard function by the relevant factor(s), fitting separate analysis models within each stratum and combining stratum-specific estimates of association using meta-analysis [10], or using the methods for analysing stratified case-cohort designs described by Borgan et al [11].

Existing STROBE guidance [7] recommends the use of Kaplan-Meier plots for a cohort study; these can also be helpful for presenting results of a case-cohort study, although Kaplan-Meier estimates need to take into account the oversampling of cases in this design [9].

### Further considerations

Most of the papers identified in our review excluded individuals with missing covariate data. This approach results in a loss of efficiency and only gives unbiased estimates if missingness can be assumed to be independent of outcome, conditional on observed covariates [12]. Some papers attempted imputation approaches, but further research is needed into how the case-cohort design should be accounted for in the imputation model, before specific recommendations can be made.

The main focus of our review was on the use of case-cohort studies to estimate associations between an exposure and an outcome, rather than to develop and evaluate risk prediction models (although such papers were not excluded from the scope). Following recent publication of two papers describing adaptations to the case-cohort setting of standard measures of risk prediction [13], [14], it seems likely that more papers on risk prediction will appear in future. Our recommendations for reporting (above) would still apply; a clear description of the subcohort sampling fraction and how it was calculated would be particularly important given its pivotal role in these methods.

Despite the fact that the case-cohort design was first proposed nearly 30 years ago, it is still relatively uncommon compared with other observational study designs, and specific issues related to design and analysis are likely to be less well known to the majority of researchers. Our review suggests that in recent years the use of case-cohort studies appears to be increasing, and therefore we hope our recommendations, which are summarized in Table 3, will help authors, reviewers and editors to achieve greater consistency and quality in how they are reported in the future.

**Table 3:** Summary of recommendations for reporting case-cohort studies, to be used alongside existing STROBE guidelines.

Topic	Recommendation
Study design/participants	Define how the random subcohort was selected, including information on the size of the original cohort, the subcohort sampling fraction, stratification factors (if relevant), and exclusion criteria that were applied either to the original cohort before sampling or to the subcohort before analysis.
Descriptiveinformation	Summarize baseline characteristics of cases/non-cases, cases/subcohort or exposure groups within the subcohort; summarized characteristics of the original cohort are also helpful.
Statistical methods(general)	Describe how statistical methods have accounted for the oversampling of cases in the study design, including details of how individuals have been weighted in the analysis.
Statistical methods(stratified design)	If a stratified design has been used, describe how the stratifying factors have been accounted for in the statistical analysis.
Statistical methods(model assumptions)	If a weighted Cox proportional hazards model has been used, describe how the proportional hazards assumption of the model was assessed.

## Supporting Information

Appendix S1 **List of journals/databases included in the literature search.**
(DOCX)

Appendix S2 **References of 32 papers included in review.**
(DOCX)
