# Supplementary material for: A Review of Published Analyses of Case-Cohort Studies and Recommendations for Future Reporting
Source: PLoS One. 2014 Jun 27;9(6):e101176. doi: 10.1371/journal.pone.0101176 (PMC4074158; doi:10.1371/journal.pone.0101176)
Supplement: Appendix S1 — List of journals/databases included in the literature search. (DOCX) [file pone.0101176.s001.docx]

**Appendix S1.**

The following journals were searched and yielded the number of papers given in parentheses after each journal name: PLoS One (15 papers), American Journal of Epidemiology (6 papers), PLoS Medicine (3 papers), International Journal of Epidemiology (2 papers), Journal of Internal Medicine (2 papers), Journal of the American Medical Association (2 papers), BMJ Open (1 paper), European Journal of Epidemiology (1 paper).

The following journals/databases were also searched, but did not yield any papers meeting the search criteria: American Journal of Medicine, American Journal of Preventive Medicine, Annals of Family Medicine, Annals of Internal Medicine, Archives of Internal Medicine, BMC Medicine, BMJ, British Medical Bulletin, Canadian Medical Association Journal, Cochrane database of systematic reviews, Epidemiology, Journal of Clinical Epidemiology, Lancet, Mayo Clinic Proceedings, Medicine, New England Journal of Medicine.
