# Supplementary material for: A Review of Published Analyses of Case-Cohort Studies and Recommendations for Future Reporting
Source: PLoS One. 2014 Jun 27;9(6):e101176. doi: 10.1371/journal.pone.0101176 (PMC4074158; doi:10.1371/journal.pone.0101176)
Supplement: Appendix S2 — References of 32 papers included in review. (DOCX) [file pone.0101176.s002.docx]

**Appendix S2.**

1. Abbasi A, Bakker SJ, Corpeleijn E, van der AD, Gansevoort RT, et al. (2012) Liver function tests and risk prediction of incident type 2 diabetes: evaluation in two independent cohorts. PLoS One 7: e51496.

2. Beulens JW, van der Schouw YT, Bergmann MM, Rohrmann S, Schulze MB, et al. (2012) Alcohol consumption and risk of type 2 diabetes in European men and women: influence of beverage type and body sizeThe EPIC-InterAct study. J Intern Med 272: 358-370.

3. Bohnert AS, Valenstein M, Bair MJ, Ganoczy D, McCarthy JF, et al. (2011) Association between opioid prescribing patterns and opioid overdose-related deaths. JAMA 305: 1315-1321.

4. Braem MG, Onland-Moret NC, van den Brandt PA, Goldbohm RA, Peeters PH, et al. (2010) Reproductive and hormonal factors in association with ovarian cancer in the Netherlands cohort study. Am J Epidemiol 172: 1181-1189.

5. Cole SR, Hudgens MG, Tien PC, Anastos K, Kingsley L, et al. (2012) Marginal structural models for case-cohort study designs to estimate the association of antiretroviral therapy initiation with incident AIDS or death. Am J Epidemiol 175: 381-390.

6. de Jong PA, Gondrie MJ, Buckens CF, Jacobs PC, Mali WP, et al. (2011) Prediction of cardiovascular events by using non-vascular findings on routine chest CT. PLoS One 6: e26036.

7. Drogan D, Sheldrick AJ, Schutze M, Knuppel S, Andersohn F, et al. (2012) Alcohol consumption, genetic variants in alcohol deydrogenases, and risk of cardiovascular diseases: a prospective study and meta-analysis. PLoS One 7: e32176.

8. Du H, Vimaleswaran KS, Angquist L, Hansen RD, van der AD, et al. (2011) Genetic polymorphisms in the hypothalamic pathway in relation to subsequent weight change--the DiOGenes study. PLoS One 6: e17436.

9. Herder C, Baumert J, Zierer A, Roden M, Meisinger C, et al. (2011) Immunological and cardiometabolic risk factors in the prediction of type 2 diabetes and coronary events: MONICA/KORA Augsburg case-cohort study. PLoS One 6: e19852.

10. Herder C, Peeters W, Illig T, Baumert J, de Kleijn DP, et al. (2011) RANTES/CCL5 and risk for coronary events: results from the MONICA/KORA Augsburg case-cohort, Athero-Express and CARDIoGRAM studies. PLoS One 6: e25734.

11. Hughes LA, Simons CC, van den Brandt PA, Goldbohm RA, van Engeland M, et al. (2011) Body size and colorectal cancer risk after 16.3 years of follow-up: an analysis from the Netherlands Cohort Study. Am J Epidemiol 174: 1127-1139.

12. Hughes LA, van den Brandt PA, Goldbohm RA, de Goeij AF, de Bruine AP, et al. (2010) Childhood and adolescent energy restriction and subsequent colorectal cancer risk: results from the Netherlands Cohort Study. Int J Epidemiol 39: 1333-1344.

13. Hughes MF, Saarela O, Stritzke J, Kee F, Silander K, et al. (2012) Genetic markers enhance coronary risk prediction in men: the MORGAM prospective cohorts. PLoS One 7: e40922.

14. Huxley RR, Lopez FL, MacLehose RF, Eckfeldt JH, Couper D, et al. (2013) Novel association between plasma matrix metalloproteinase-9 and risk of incident atrial fibrillation in a case-cohort study: the Atherosclerosis Risk in Communities study. PLoS One 8: e59052.

15. InterAct (2012) Tea consumption and incidence of type 2 diabetes in Europe: the EPIC-InterAct case-cohort study. PLoS One 7: e36910.

16. Juraschek SP, Shantha GP, Chu AY, Miller ER, 3rd, Guallar E, et al. (2013) Lactate and risk of incident diabetes in a case-cohort of the atherosclerosis risk in communities (ARIC) study. PLoS One 8: e55113.

17. Karakas M, Koenig W, Zierer A, Herder C, Rottbauer W, et al. (2012) Myeloperoxidase is associated with incident coronary heart disease independently of traditional risk factors: results from the MONICA/KORA Augsburg study. J Intern Med 271: 43-50.

18. Keszei AP, Schouten LJ, Goldbohm RA, van den Brandt PA (2010) Dairy intake and the risk of bladder cancer in the Netherlands Cohort Study on Diet and Cancer. Am J Epidemiol 171: 436-446.

19. Lamb MM, Simpson MD, Seifert J, Scott FW, Rewers M, et al. (2013) The association between IgG4 antibodies to dietary factors, islet autoimmunity and type 1 diabetes: the Diabetes Autoimmunity Study in the Young. PLoS One 8: e57936.

20. Langenberg C, Sharp SJ, Schulze MB, Rolandsson O, Overvad K, et al. (2012) Long-term risk of incident type 2 diabetes and measures of overall and regional obesity: the EPIC-InterAct case-cohort study. PLoS Med 9: e1001230.

21. Lu Y, Vaarhorst A, Merry AH, Dolle ME, Hovenier R, et al. (2012) Markers of endogenous desaturase activity and risk of coronary heart disease in the CAREMA cohort study. PLoS One 7: e41681.

22. Montonen J, Drogan D, Joost HG, Boeing H, Fritsche A, et al. (2011) Estimation of the contribution of biomarkers of different metabolic pathways to risk of type 2 diabetes. Eur J Epidemiol 26: 29-38.

23. Pfister R, Sharp S, Luben R, Welsh P, Barroso I, et al. (2012) Mendelian randomization study of B-type natriuretic peptide and type 2 diabetes: evidence of causal association from population studies. PLoS Med 8: e1001112.

24. Sacerdote C, Ricceri F, Rolandsson O, Baldi I, Chirlaque MD, et al. (2012) Lower educational level is a predictor of incident type 2 diabetes in European countries: The EPIC-InterAct study. Int J Epidemiol 41: 1162-1173.

25. Sartorius T, Staiger H, Ketterer C, Heni M, Machicao F, et al. (2012) Association of common genetic variants in the MAP4K4 locus with prediabetic traits in humans. PLoS One 7: e47647.

26. Schouten LJ, van Dijk BA, Lumey LH, Goldbohm RA, van den Brandt PA (2011) Energy restriction during childhood and early adulthood and ovarian cancer risk. PLoS One 6: e27960.

27. Schrijvers EM, Koudstaal PJ, Hofman A, Breteler MM (2011) Plasma clustering and the risk of Alzheimer disease. JAMA 305: 1322-1326.

28. Simons CC, Hughes LA, van Engeland M, Goldbohm RA, van den Brandt PA, et al. (2013) Physical activity, occupational sitting time, and colorectal cancer risk in the Netherlands cohort study. Am J Epidemiol 177: 514-530.

29. Simons CC, Schouten LJ, Weijenberg MP, Goldbohm RA, van den Brandt PA (2010) Bowel movement and constipation frequencies and the risk of colorectal cancer among men in the Netherlands Cohort Study on Diet and Cancer. Am J Epidemiol 172: 1404-1414.

30. Sinner MF, Reinhard W, Muller M, Beckmann BM, Martens E, et al. (2010) Association of early repolarization pattern on ECG with risk of cardiac and all-cause mortality: a population-based prospective cohort study (MONICA/KORA). PLoS Med 7: e1000314.

31. Stegger JG, Schmidt EB, Tjonneland A, Kopp TI, Sorensen TI, et al. (2012) Single nucleotide polymorphisms in IL1B and the risk of acute coronary syndrome: a Danish case-cohort study. PLoS One 7: e36829.

32. Wennberg P, Rolandsson O, van der AD, Spijkerman AM, Kaaks R, et al. (2013) Self-rated health and type 2 diabetes risk in the European Prospective Investigation into Cancer and Nutrition-InterAct study: a case-cohort study. BMJ Open 3: e002436.
